# Automatic Image Analysis Method as a Tool to Evaluate the Anisotropy of Autoclaved Aerated Concrete for Moisture and Heat Transport

**DOI:** 10.3390/ma17194903

**Published:** 2024-10-07

**Authors:** Dariusz Majerek, Elżbieta Sędzielewska, Magdalena Paśnikowska-Łukaszuk, Ewa Łazuka, Zbigniew Suchorab, Grzegorz Łagód

**Affiliations:** 1 Faculty of Mathematics and Information Technology, Lublin University of Technology, Nadbystrzycka 38, 20-618 Lublin, Poland; d.majerek@pollub.pl (D.M.); m.pasnikowska-lukaszuk@pollub.pl (M.P.-Ł.); e.lazuka@pollub.pl (E.Ł.); 2 Faculty of Environmental Engineering, Lublin University of Technology, Nadbystrzycka 40B, 20-618 Lublin, Poland; z.suchorab@pollub.pl

**Keywords:** measurement methods, image analysis, building materials, material anisotropy, moisture transport, heat transfer, thermal conductivity

## Abstract

In this article, the results of studies testing the anisotropy of autoclaved aerated concrete in terms of water and heat transport are presented. Using image analysis techniques, a study was conducted on four different samples of concrete produced in the same process. To ensure the comparability of results, the pictures were taken from a fixed distance with the same lens settings trimmed to a set size. Cross-sectional profiles of the material were examined and were arranged in two directions: perpendicular and parallel to the growth direction occurring in the autoclave. For each block, approximately 4750 objects were obtained, with an average of 2700 objects along the wall and 2050 across it. As a result of the comparative analysis, metrics concerning pores, significantly distinguishing the profile direction, were identified. These included the pore area (area), the maximum and minimum distance between points on the perimeter (Feret, MinFeret), lengths of the major and minor axes of the fitted ellipse (major, minor), and the ratio of the area of selection to its convex hull (solidity). As a reference, standard investigations were conducted for moisture transport using the time domain reflectometry setup and for thermal conductivity values using the steady-state heat flow plate apparatus.

## 1. Introduction

Currently, in the construction industry, it is essential to ensure that energy usage in buildings is highly efficient [1,2]. This requires using the materials characterized by favorable thermal properties, e.g., aerated concrete or clay brick. Autoclaved aerated concrete, also known as AAC, is a commonly used construction material [3].

Autoclaved aerated concrete (AAC) blocks with low thermal conductivity and high heat resistance can serve simultaneously as the building’s wall and insulation [4]. Aerated concrete is a mortar with pulverized sand and industrial waste like fly ash as a filler, in which air is entrapped artificially by chemical or mechanical means, resulting in a significant reduction in density [5]. Apart from thermal properties, the moisture content of partitions is an important parameter as far as building operation is concerned. Therefore, studying the saturation or absorption capacity of the most popular materials is of great importance. The high moisture content rapidly increases the freeze–thaw deterioration of concrete [6,7]. The heat transfer and moisture transport in porous media are highly coupled and not equivalent, i.e., the heat transfer has a greater effect on moisture transport, while the moisture transport has a smaller effect on heat transfer [8]. Problems for concrete structures are closely related to temperature and humidity fields in concrete [9]. The main factors determining the rate of moisture transport in concrete include temperature, relative humidity (RH), microstructure, and the porosity of concrete [10].

The AAC generally exhibits a homogenous general distribution of pores and dimensions thereof. Nevertheless, the irregular positioning of pores may occur in the course of manufacturing [11]. Thus, the water transport rate could be affected. The direction in which the block is placed in the wall affects the flow of water and, to some extent, the flow of heat, which means that the way the block is placed can affect the faster capillary flow of water up the partition or crosswise to the wall. Due to the porosity configuration of the AAC, the thermal and mechanical anisotropy were investigated [12,13,14,15].

Moisture content can be tested using various methods, with the gravimetric method being the standard one; it consists of sample weighing prior to and following drying [16]. Nevertheless, a sample has to be collected before using this method, so it is considered invasive. Alternatively, moisture detection can be carried out using indirect methods, which involve measuring the physical values governed by moisture [17]. These include measuring the value of apparent permittivity, as well as the resistance of the electrical current [18].

Apparent permittivity (ε) constitutes a measure of matter particles behavior in an alternating electric field; thus, it is higher in the case of asymmetric molecules of water (ε_water_ = 80) compared to solid phase (ε_solid_ = 3–8) or air (ε_air_ = 1) [19,20,21]. In the case of moist, porous materials, it is possible to measure apparent permittivity by means of three methods, namely time domain reflectometry (TDR) [22], capacitive sensors (FD), or microwave antennas (MW) [19,23,24,25]. TDR employ electromagnetic waves to analyze material properties within a medium and are widely utilized for determining moisture content [26]. TDR is characterized by the ability to accurately measure the electrical permittivity of a material and the fact that there is a good relationship between the material’s permittivity and its water content [27,28]. The application of TDR methods to determine *θ_v_* is based on the measurement of the travel time of an electromagnetic wave pulse (usually <1.5 GHz) generated by a TDR cable tester through a waveguide called a probe or sensor, which is inserted into porous material [25]. The TDR technique is commonly used for moisture content measurements in soil but is considered a relatively new method with regard to its application in construction materials [27].

The anisotropy of water transport is a consequence of the shape and distribution of pores. The water rise rate is probably dependent upon growth direction [13]. Porosity is considered when assessing porous surfaces [29], soil [30], thermally sprayed coatings [31], sintering [32], and clastic rocks [33,34].

Moreover, pore characteristics may be linked to the physical properties characterizing materials, including frost resistance [35,36] and the compressive strength of cement mortars [37].

This paper proposes using image analysis for the assessment of water capillary uptake and thermal conductivity coefficient. The AAC specimen images were analyzed to determine the pore features and their impact on the rise rate, including minimum and maximum Feret’s diameter, length, circumference, etc. [38].

## 2. Materials and Methods

The study was carried out on cuboid AAC blocks with a density equal to 400 kg/m^3^. The adopted assumption was that the AAC is anisotropic as a result of the material expansion and curing process during material production. Aerated concrete is produced from quartz sand, acting as a microaggregate alongside cement, lime, gypsum, aluminum powder, and water. The most important stage in the production of aerated concrete is its growth and maturation. An important role in these processes is played by aluminum powder, which causes the mass to grow in the appropriate conditions at a high pH of the mixture, which is ensured by the presence of calcium hydroxide. In the reaction of aluminum powder with calcium hydroxide, hydrogen is released, which causes the mass to grow, as a result of which gas bubbles occur in the structure of the material mixture, which consequently creates a foamy mixture growing in the vertical direction. The hydrogen then escapes and is replaced by air, which fills the empty spaces of the aerated concrete. That is why the pore shape differs for perpendicular and parallel directions in material specimens depending on the expansion process. During expansion and curing, therefore, the processes of moisture migration, as well as thermal parameters, may vary. Hence, the considered samples were cut in both directions, i.e., along and perpendicular to the expansion and curing direction. Samples perpendicular to the expansion and curing direction are marked as H (horizontal direction), and samples parallel to the expansion and curing process are marked as V (vertical). The sample images were taken at a fixed distance using the same settings of the camera.

### 2.1. Moisture Migration Test Setup

The capillary action experiment shows how the sample orientation affects the dynamics of the water transport process in the sample. It is shown that the anisotropic nature of the material has an impact on this process. A moisture migration test was conducted using TDR equipment manufactured by ETest, Lublin, Poland. The laboratory setup consisted of FP/mts probes and an LOM multimeter also manufactured by ETest, Lublin, Poland.

For the experimental water migration test, four samples were prepared. The samples had a cubic shape with an edge length of 240 mm. Two of the samples are marked H1 and H2 (horizontal direction), and two of the samples are marked V1 and V2 (vertical direction). The basic parameters of each sample are presented in Table 1.

The process of capillary uptake was conducted for a period of 7 days. After the absorption experiment was finished, the samples were taken from the TDR sensors and weighed to compare reflectometric with gravimetric readouts. The test was repeated two times for horizontally cut samples (H1 and H2) and vertically cut samples (V1 and V2). The TDR method is used for porous building materials, and the precision is comparable to the classical method of capillary rise measurements according to EN 1925 [31,39].

In all the samples, the FP/mts probes were installed 50 and 100 mm above the surface of the water. Time intervals between each measurement were set for 120 s.

The laboratory conditions for the experiment included relative air humidity at 5% ± 10% and a constant temperature of 20 °C ± 1 °C.

### 2.2. Thermal Conductivity Test Setup

The determination of thermal conductivity depending on the direction of heat flow in the material sample allows for the demonstration of the anisotropy of aerated concrete for thermal parameters. For that aim, samples of material were prepared and cut in three dimensions. An experiment to determine the thermal conductivity coefficient was conducted using the stationary method and heat flow meter FOX 314 manufactured by TA Instruments, New Castle, USA; each experiment was repeated three times for both directions. Samples for the FOX heat flow meter were cut into the following dimensions: 240 mm × 240 mm × 100 mm, which was determined by the dimensions of aerated concrete blocks (240 and 240) and the dimensions of the measuring chamber of the heat flow meter (300 mm × 300 mm × 100 mm). For this aim, a polystyrene frame was prepared to cover the aerated concrete and prevent uncontrolled heat flow on the sides.

Four samples cut perpendicularly to the expansion and curing process were marked as H1, H2, H3, and H4. On the other hand, the samples cut parallel were marked as V1, V2, V3, and V4.

The measurement temperatures were set for 10 °C (cold side) and 30 °C (warm side) to achieve an average measurement temperature equal to 20 °C.

### 2.3. Image Analysis

To analyze the pore structure and its influence on water absorption in the AAC, a series of image processing and analysis steps were performed using Fiji v2.9.0/1.53t software (a distribution of ImageJ) [40]. The steps were structured to ensure a robust and repeatable analysis process. In this enhanced methodology, additional techniques and metrics were incorporated to capture a more comprehensive understanding of the pore geometry, anisotropy, and its influence on capillary action. The primary objectives of the image analysis were to (1) extract pore-related features that characterize the anisotropic nature of the material and (2) quantify the directional differences in pore morphology between samples oriented along and across the growth direction.

**Figure 1:**
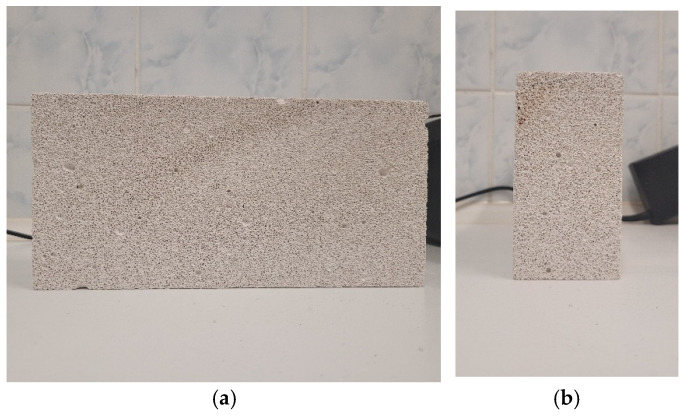
Positions of the sample for which image taken (without zooming in): (**a**) along the direction of growth (growth from right to left); (**b**) across the direction of growth.

**Figure 2:**
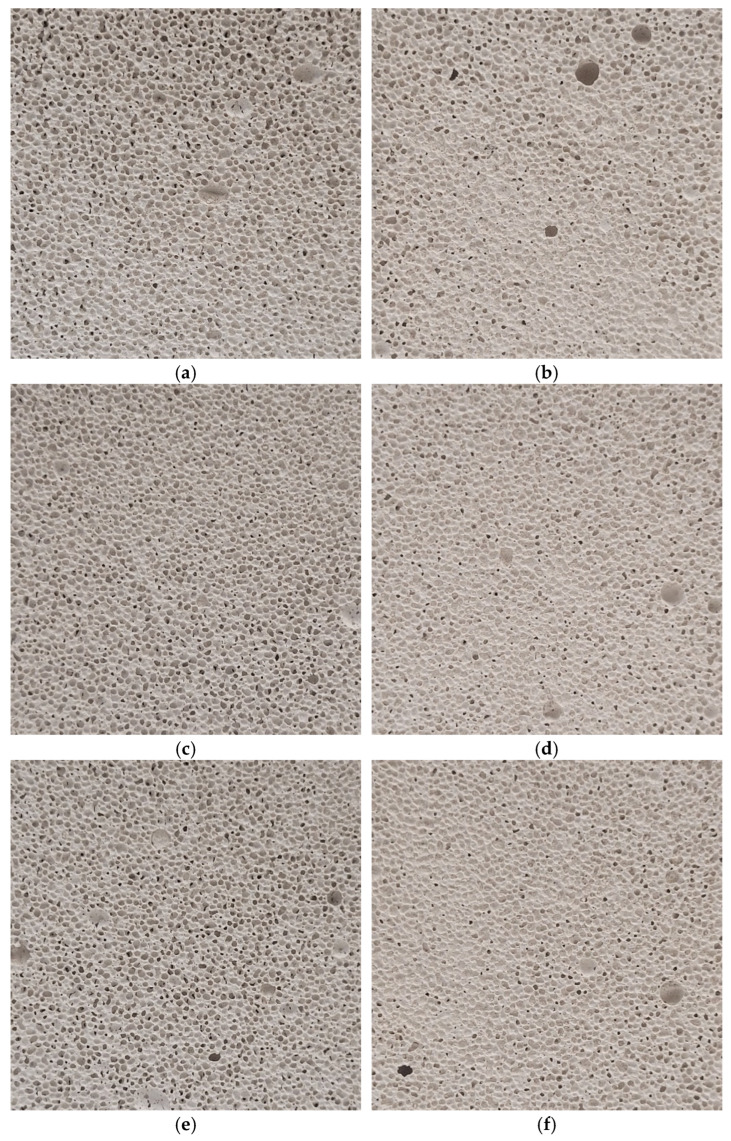

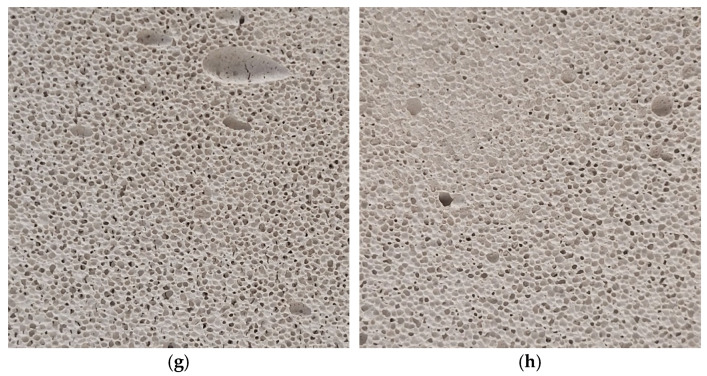
(**a**–**h**) Images were cropped to 2300 × 2300 px. The cross-section along the expansion and curing direction and perpendicular to expansion and curing direction are shown on the left-hand and right-hand side, respectively, for all four samples in sequence.

High-resolution images of AAC samples were acquired using a fixed camera setup to ensure consistency in pixel size and resolution across all images. The images were taken for four samples in both vertical (V) and horizontal (H) orientations corresponding to the expansion and curing directions of the concrete blocks, as shown in Figure 1. Each image was trimmed to 2300 × 2300 pixels to eliminate edge effects and to maintain uniformity across the different samples. This made it possible to enlarge the test sample relative to that in [41]. In addition, the study was performed for four different samples. Figure 2 shows all the resulting images used subsequently for analysis.

The preprocessing stage was critical in ensuring that the images were optimized for accurate and efficient pore analysis. This phase involved a series of steps designed to prepare the raw image data for further processing, removing unwanted artifacts and enhancing the visibility of relevant features, such as pores, in the AAC samples.

The first step in preprocessing was grayscale conversion, where all the acquired images were converted to an 8-bit grayscale format. This step reduced the complexity of the image data by simplifying the color information, leaving only intensity values for each pixel. The conversion was essential in focusing the analysis on pore detection, as color information was unnecessary for this task. By reducing the data’s dimensionality, computational efficiency was improved without sacrificing the ability to discern pore structures.

Next, noise reduction was performed using a Gaussian blur filter [42,43]. This technique smoothed the image by reducing the high-frequency variations in pixel intensity, effectively removing noise and small artifacts that could have been misinterpreted as pores. The application of Gaussian blur ensured that only the significant structural features of the pores were retained, making the subsequent analysis more reliable. This step was crucial for avoiding the misclassification of minor imperfections, such as surface irregularities, which could skew the analysis if left untreated.

After noise reduction, thresholding [44] was applied to distinguish the pores from the surrounding solid material. Huang’s method [45] was chosen for automatic thresholding due to its ability to handle variations in image brightness and contrast, offering a balanced separation of the foreground (pores) and background (solid material). The method was carefully calibrated to optimize the differentiation between the pores and the rest of the material. While alternative thresholding techniques, such as Otsu’s [46] and Li’s [47] methods, were evaluated, Huang’s method consistently produced the best results across multiple samples. This consistency was particularly important given the structural complexity and variability of the AAC samples, ensuring the reliable extraction of pore regions.

Once the thresholding process was completed, the next stage involved the segmentation of the detected pore regions. Segmentation is the process of partitioning the image into distinct regions that correspond to individual pores. For this purpose, the watershed algorithm [48] was employed, which is particularly effective at separating closely adjacent pores that might otherwise be merged due to proximity. The watershed algorithm uses gradient information from the image to detect boundaries between touching pores, ensuring that each pore is treated as an individual object [48].

After segmentation, object detection was performed, wherein each pore was identified as a distinct object within the image. The geometric properties of each detected pore, such as area, perimeter, and shape descriptors, were then extracted for further analysis. These features provided valuable insights into the structure and distribution of the pores, which were critical for understanding the anisotropic nature of the AAC material. To maintain the accuracy of the analysis, the segmented objects were manually reviewed. This manual inspection ensured that the automatic detection process had correctly identified and separated pores without merging or splitting them incorrectly, especially in cases where pore boundaries were less distinct.

**Figure 3:**
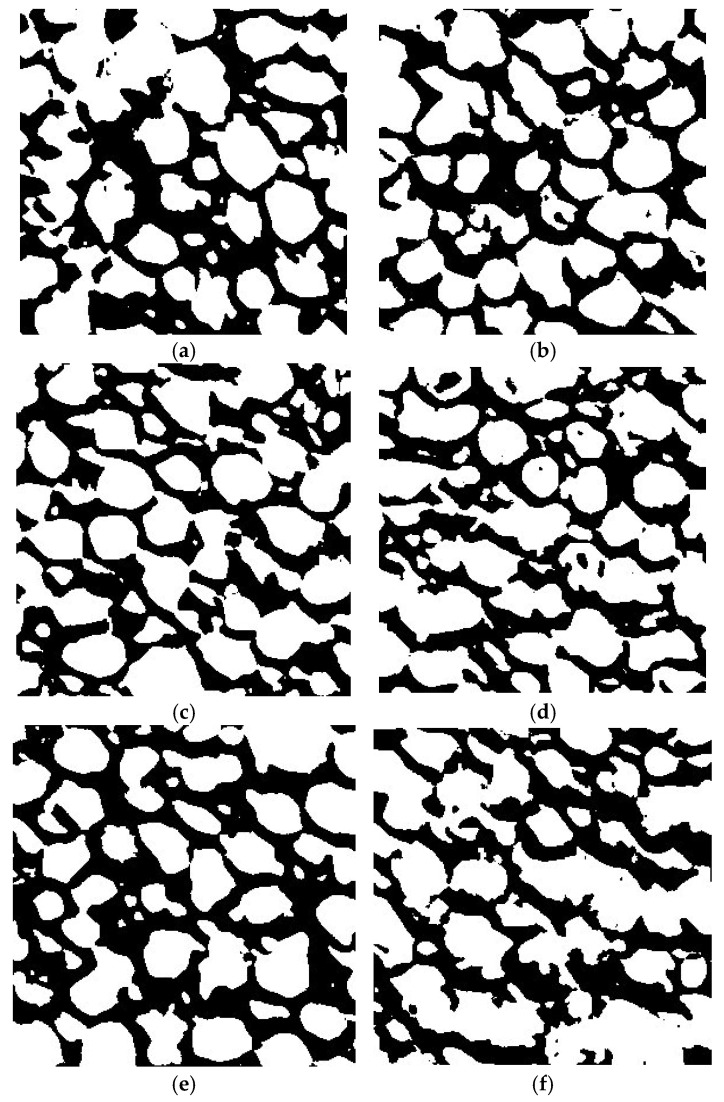

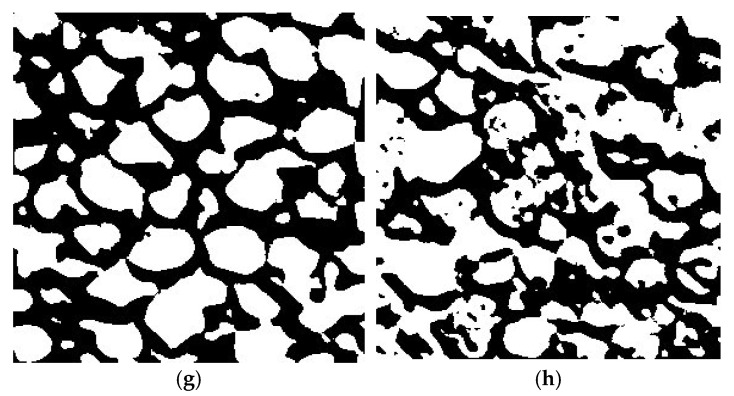
(**a**–**h**) Images following thresholding using Huang’s method at 200% zoom.

By combining advanced segmentation techniques with careful manual verification, the process ensured that pore detection was both accurate and consistent across all samples, providing a solid foundation for subsequent statistical and geometric analysis. Figure 3a–h show the results.

The analyzed elements yielded the following measures:

- Angle—(0–180 degrees) constitutes the angle between the major axis and a line parallel to the horizontal axis of the image.
- Major—the primary axis of the best-fitting ellipse
- Minor—the secondary axis of the best-fitting ellipse.
- Area—the selected area in square pixels.
- Perimeter—the length of the external border of the selection.
- Circ.—the shape factor, 1.0, denotes that the object is a circle, while 0.0 is an elongated shape; it is expressed using the equation below:

(1) $$\mathrm{circularity}=\frac{4\pi \cdot \mathrm{area}}{{\mathrm{perimeter}}^{2}}.$$

- AR—the ratio of the axis length of the ellipse fitted to the shape of the selection.
- Round—roundness, expressed using the following equation:
  (2) $$\mathrm{round}=\frac{4\cdot \mathrm{area}}{\pi \cdot {{\mathrm{major}}_{\mathrm{axis}}}^{2}},$$
  where ${\mathrm{major}}_{\mathrm{axis}}$ denotes the major axis of the ellipse fitted to the selection.
- Feret’s diameter—the largest distance between two points along the border of the selection.
- Feret’s angle—(0–180 degrees) the angle between Feret’s diameter and the line, which is parallel to the horizontal axis of the image.
- MinFeret—the smallest distance between two points along the boundary of the selection.
- Solidity—the ratio of the selected area to the convex hull.

Statistical analysis was then conducted to compare the pore characteristics between horizontal and vertical orientations. Descriptive statistics, such as the mean and median, were calculated for each metric, and distribution analysis was performed using histograms and kernel density estimates [49,50]. The Wilcoxon–Mann–Whitney test [43] was used to assess significant differences between the two orientations. An anisotropy index [51], based on the ratio of the major to minor axis, was calculated to quantify the degree of anisotropy.

Finally, the extracted pore metrics were correlated with capillary uptake data from time-domain reflectometry (TDR) measurements. This correlation helped identify which pore features—such as area, solidity, or Feret’s diameter—were most strongly associated with water absorption rates. This approach provided a direct link between pore geometry and material performance, offering valuable insights into the anisotropic behavior of AAC and its impact on moisture transport.

## 3. Results

### 3.1. Results on Moisture Migration Experiment

Data from TDR readouts were acquired as a set of apparent permittivity values (ε) and then recalculated into moisture and volumetric water content, which is a standard measure used in the determination of moisture. To recalculate apparent permittivity into moisture, the Malicki model was applied [52]:

(3) $$\theta =\frac{\left(\varepsilon^{0.5}-0.819-0.168\rho -0.159\rho^{2}\right)}{7.17+1.18\rho },$$

where ε—apparent permittivity read by the TDR equipment; ρ—density of tested material in dry conditions [g/cm^3^].

**Figure 4:**
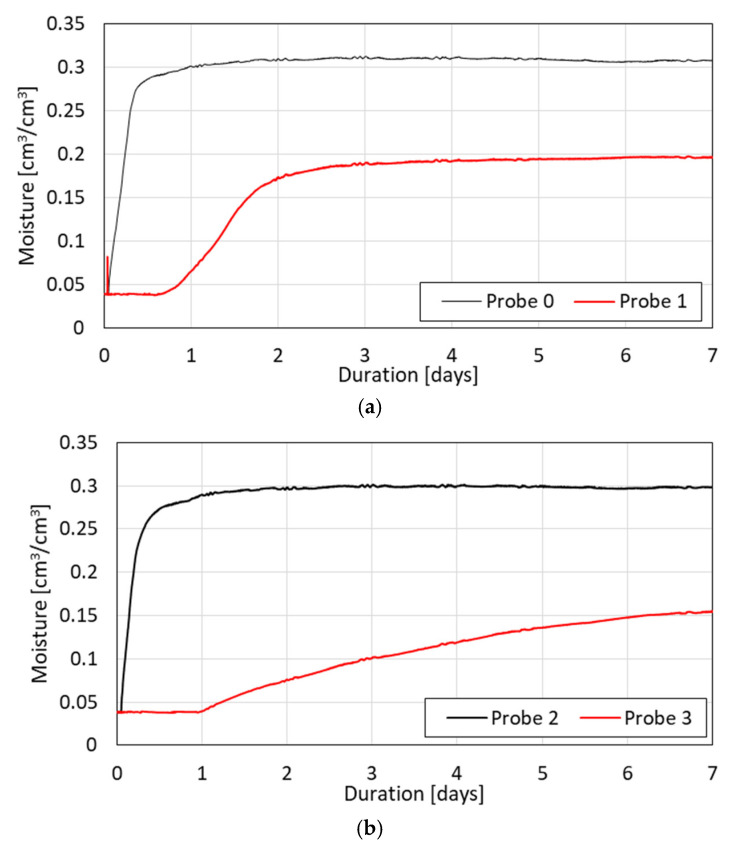

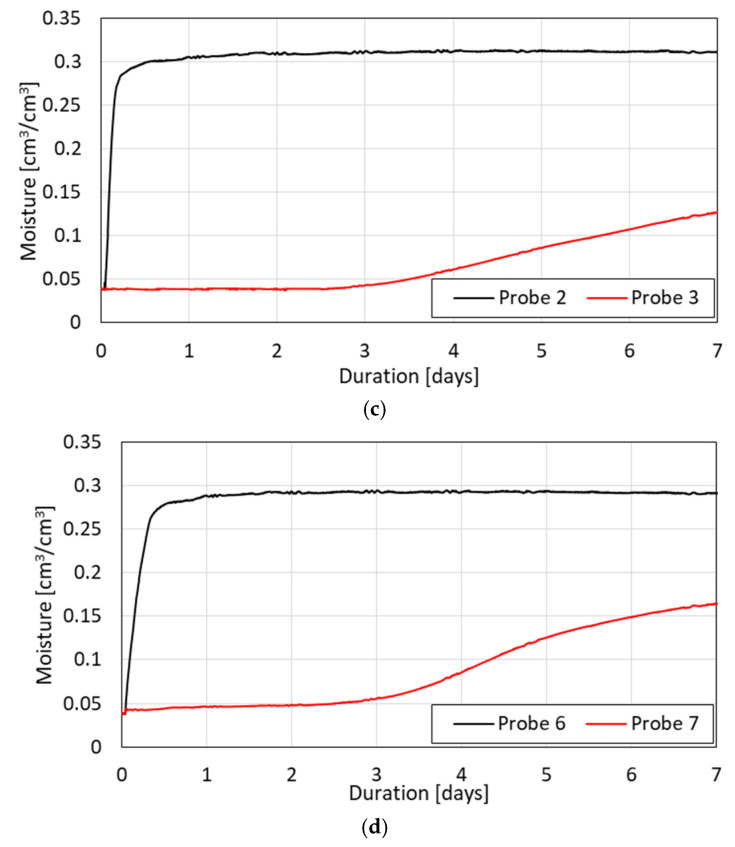
(**a**–**d**) Capillary uptake process (absorption) in two types of samples, V and H. (**a**) V1 sample—absorption process; (**b**) V2 sample—absorption process; (**c**) H1 sample—absorption process; and (**d**) H2 sample—absorption process.

Figure 4 shows the visible changes in moisture captured via the TDR equipment for the absorption process.

The diagrams show that the capillary uptake process differed for both sample types. In the case of all samples, the presence of water at the level of 0.05 cm^3^/cm^3^ was noticed initially. For samples V1 and V2, moisture readouts on the upper TDR probe (Probe 1) were found 50 mm above the bottom. Probe 0 appeared on the first day of the experiment, stabilizing from the third day onward. In turn, samples H1 and H2 exhibited readouts at the upper probe starting from the third day and did not stabilize even after 8 days.

The mass of samples for moisture evaluation experiment before and after the session is presented in Table 2.

### 3.2. Results on Thermal Conductivity Coefficient Evaluation

The readouts of thermal conductivity coefficients are presented in Table 3.

As can be seen, the values presented in Table 3 are not significantly different for samples of the V and H types. It can be noticed that the average value of the thermal conductivity coefficient for V-type samples equals 0.1143 W/mK while, in the case of H-type samples, it is equal to 0.1165 W/mK, and the difference is only 0.0022 W/mK.

### 3.3. Result of Image Processing

**Figure 5:**
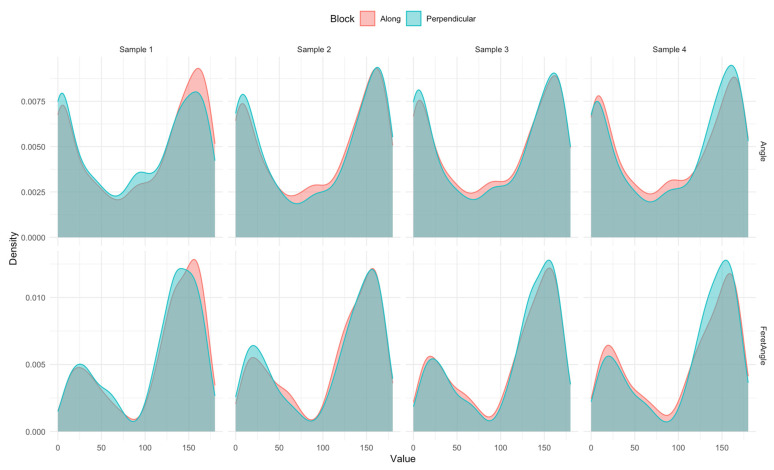
Distributions of angle and Feret angle variables by block setting for all four samples.

**Figure 6:**
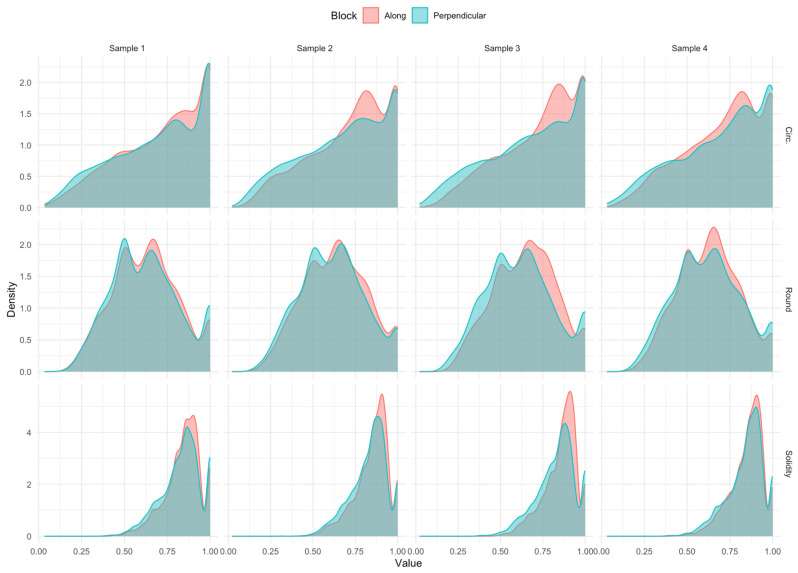
Distributions of circ., round and solidity variables by block setting for all four samples.

The number of pores in samples used for comparative analysis was above 3000 for the sample along the growth direction, as well as perpendicular (see Table 4). The distribution of variables was examined first. As far as the shape is concerned, there are similarities in distribution. However, the intensity of observations is different depending on the block direction. Figure 5 and Figure 6 show the distribution. Figure 5 presents the densities of the metrics for the pore slope; here, the differences do not appear significant except for sample 1. Figure 6 shows the metrics for assessing the pore roundness. The solidity metric significantly distinguishes the direction in all four samples.

Comparative tests were carried out to determine the significance of differences. The Wilcoxon–Mann–Whitney test [53] was used for this purpose since the majority of distributions were not normal. When the shapes of distributions were similar, the hypothesis that the medians of two groups were equal was verified. Such statistics were chosen because distributions are asymmetric. Area, major, minor, Feret, MinFeret, and solidity were found to be significantly different in all four samples from the measures described in the previous section. Table 5 contains the *p*-value from the test for all the metrics, which shows which parameters differ significantly and which do not.

**Figure 7:**
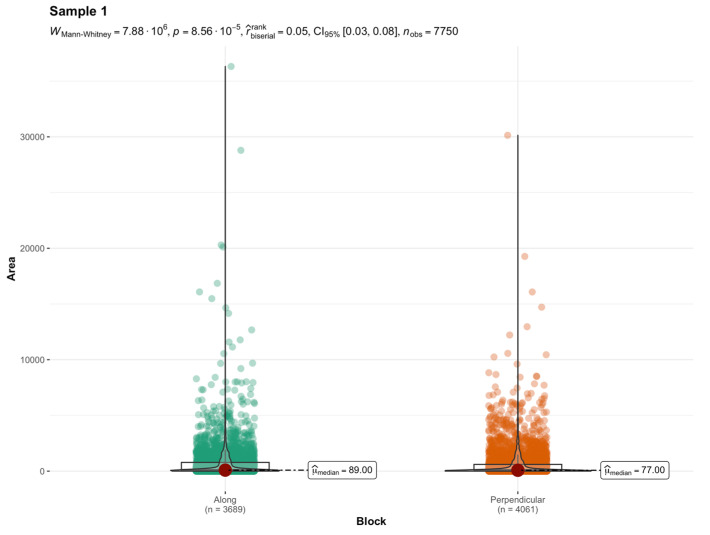
The violin diagram of the area variable and results of the equality test of medians for sample 1.

**Figure 8:**
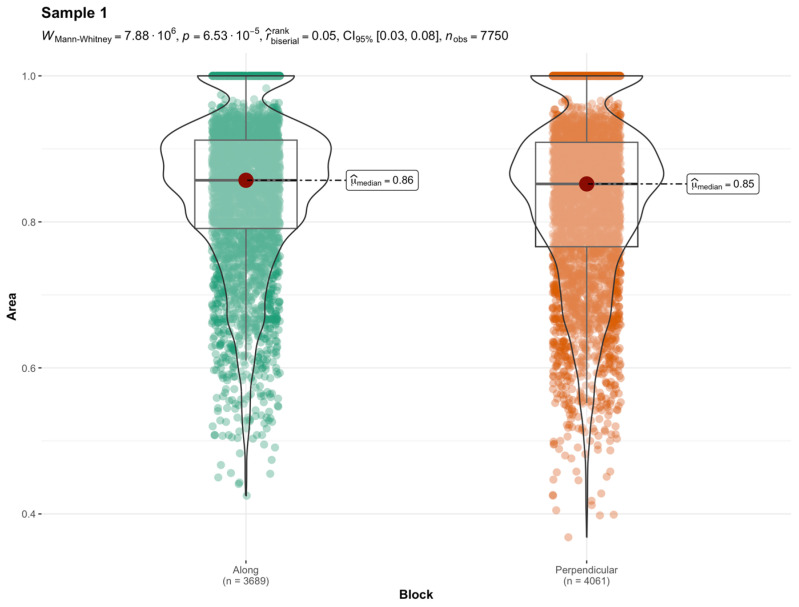
The violin diagrams of the solidity variable and results of the equality test of medians for sample 1.

Among these metrics, what significantly distinguishes the cross-section are also measures of the pore stretch length, major, minor, Feret and MinFeret, and area. The result of the median equality test for the area variable is presented in Figure 7. Figure 8, in turn, presents the median equality test for the solidity variable.

## 4. Discussion

From the conducted research, the anisotropy influence of both water and heat transport was confirmed. This was specially recognized by the TDR probe readouts, which are located 50 mm above the water level during the monitoring of the process of capillary uptake, where, in the case of samples V1 and V2, moisture appears earlier within the first day of the process, while in the case of the samples of the H type, it appears later during the third day of the process and reaches its maximum value letter. It shows that, in this case, the capillary action is more rapid in the case of samples with a vertical flow direction (V). This difference proves the anisotropic features of the tested material. Similar dependences were confirmed by an evaluation of the samples for mass moisture determined gravimetrically at the end of the experiment where, in the case of the V samples, the average increase in water equaled about 306 g while, in case of the H samples, it equaled 506 g and the difference between the two directions reached 65%.

This situation is similar in the case of the thermal conductivity coefficient, where the differences are not so visible, and the average values of the lambda coefficient are equal to 0.1143 W/mK in the case of the V samples and 0.1165 W/mK for the samples of type H. It must be remembered that this small difference could be treated as below the accuracy of the used device [54].

Research concerning the influence of material anisotropy on moisture and thermal properties has been conducted by many researchers like Zhao et al. [55], where the characterization of hygrothermal properties of two species of wood was tested for the anisotropy impact. The conducted research has shown that the difference between readouts of thermal conductivity differs by about 42% between the particular heat transport directions. It ought to be mentioned that wood is a significantly anisotropic material. Similar observations were noticed for moisture transport measured as the water absorption coefficient, where the differences reached up to 100%. In another study conducted by Zhao and Plagge [56], sandstone was tested in two different directions, and the readouts revealed no differences in thermal conductivity coefficients depending on direction or small differences reaching less than 10%. Similar observations were observed for moisture, where the differences marked in % reached between 1.2 and 6.5%.

Sample (a) exhibits a greater surface area, as well as the perimeter of pores aligned with the direction of uptake, longer diameters of Feret and MinFeret, as well as longer minor and major axes of the fitted ellipse. Moreover, samples vary in terms of the angle between the Feret diameter and horizontal axis, as well as the angle between the major axis and horizontal axis. Marked differences in Feret angle, as well as angle metrics, might show varied pore alignment. When compared to TDR results, it can be observed that in sample (b)—which has smaller pore sizes—the moisture at a 50 mm height is lower than in the case of sample (a), even though quick saturation to the height of the lower probe occurred in the same time frame.

## 5. Conclusions

This study investigates the anisotropic properties of autoclaved aerated concrete (AAC) with a focus on moisture transport and thermal conductivity. The research combines the automatic image analysis of pore structures with experimental methods to evaluate how these properties differ based on the material’s growth direction. Samples of AAC were prepared and analyzed in two orientations: vertical (V), parallel to the expansion and curing process, and horizontal (H), perpendicular to it. The primary objective was to understand how the direction of pore growth influences both moisture uptake and heat transfer.

The image analysis was performed using Fiji v2.9.0/1.53t software, where high-resolution images of the concrete samples were processed to extract key pore features, such as area, perimeter, Feret’s diameter, and solidity. These parameters were then statistically analyzed to detect significant differences between the samples oriented in the two directions. The results showed that the pore distribution is anisotropic, with significant differences in metrics like area, Feret’s diameter, and solidity between the vertical and horizontal samples.

In parallel, moisture migration experiments were conducted using time-domain reflectometry (TDR). These tests measured moisture content as water was absorbed into the samples. The results indicated that vertical samples (V) exhibited faster and more stable capillary water absorption than the horizontal samples (H), confirming the anisotropic nature of moisture transport. The correlation between the TDR results and the pore metrics from the image analysis suggests that larger and more elongated pores in the vertical direction enhance the water absorption process.

Additionally, thermal conductivity tests were performed using a stationary heat flow meter (FOX 314) manufactured by TA Instruments, New Castle, USA. The thermal conductivity measurements were relatively consistent across both sample orientations, with only minor differences between the vertical and horizontal samples. The small variations in thermal conductivity could be attributed to differences in pore structure, particularly the elongation of pores, but these variations were not large enough to be considered significant.

The combined analysis of pore structure, moisture absorption, and thermal conductivity provides valuable insights into the role of pore geometry in AAC’s anisotropic behavior. This study demonstrates that automatic image analysis can effectively capture the geometric features that influence material properties, and these findings can be directly linked to the practical performance of the material in construction, particularly in terms of moisture management and thermal efficiency. Understanding the anisotropy of AAC can help optimize its use in energy-efficient building designs, ensuring better moisture control and heat regulation.

One limitation of this approach is that the image analysis relies heavily on the resolution and quality of the images used. Any inconsistencies in image acquisition, such as variations in lighting, focus, or camera settings, can affect the accuracy of pore detection and measurement. Additionally, while image analysis provides valuable insights into pore geometry, it does not account for the full complexity of the material’s internal structure, such as potential variations in pore connectivity or the presence of microcracks, which could also influence moisture transport and thermal conductivity. Furthermore, the anisotropy observed in the samples may vary across different batches of AAC, as the manufacturing process itself can introduce variability. Another limitation is that the correlation between pore structure and material properties, such as moisture absorption and thermal conductivity, is based on surface-level observations and may not fully capture three-dimensional interactions within the bulk of the material. As a result, while image analysis provides a useful tool for understanding material behavior, it should be complemented with other techniques, such as 3D imaging or direct measurements of porosity and permeability, for a more comprehensive evaluation.

## Figures and Tables

**Table 1:** Basic physical parameters of AAC samples used in capillary uptake setup.

Sample Symbol	Dry Sample Mass [g]	Material Density [kg/m^3^]
H1	797.4	443.5
H2	793.1	442.3
V1	803.3	445.7
V2	823.5	447.4

**Table 2:** Moisture increases in tested samples.

Sample	Moist Sample Mass [g]	Moisture Increase [g]	Average Moisture Increase [g]
V1	1091.7	294.3	305.9
V2	1110.6	317.5	
H1	1313.7	510.4	505.8
H2	1324.7	501.2	

**Table 3:** Thermal conductivity coefficient determined using FOX 314 heat flow meter.

Sample	Thermal Conductivity Coefficient [W/mK]	Average Thermal Conductivity Coefficient [W/mK]
V1	0.1132	0.1143
V2	0.1138	
V3	0.1147	
V4	0.1156	
H1	0.1179	0.1165
H2	0.1163	
H3	0.1146	
H4	0.1171	

**Table 4:** The number of pores in the samples depending on the direction.

	Along	Perpendicular
Sample 1	3689	4061
Sample 2	3579	3406
Sample 3	3804	3539
Sample 4	3614	3152

**Table 5:** *p*-values of the Wilcoxon–Mann–Whitney test of comparisons for individual metrics between readings taken along and perpendicular to the direction of AAC growth.

	Sample 1	Sample 2	Sample 3	Sample 4
Area	0.00008	0.00153	0.00000	0.00118
Perim.	0.00385	0.05584	0.00000	0.00649
Major	0.00017	0.00604	0.00000	0.00435
Minor	0.00002	0.00023	0.00000	0.00019
Angle	0.00000	0.84476	0.25822	0.29619
Circ.	0.05254	0.00006	0.00000	0.95649
Feret	0.00073	0.02388	0.00000	0.00724
FeretAngle	0.00003	0.61706	0.04282	0.04874
MinFeret	0.00046	0.00352	0.00000	0.00058
AR	0.27288	0.00010	0.00000	0.05696
Round	0.27172	0.00010	0.00000	0.05691
Solidity	0.00006	0.00000	0.00000	0.00909

## Data Availability

The original contributions presented in the study are included in the article, further inquiries can be directed to the corresponding authors.
